# Nrf2 Negatively Regulates Type I Interferon Responses and Increases Susceptibility to Herpes Genital Infection in Mice

**DOI:** 10.3389/fimmu.2019.02101

**Published:** 2019-09-06

**Authors:** Camilla Gunderstofte, Marie Beck Iversen, Suraj Peri, Anne Thielke, Siddharth Balachandran, Christian Kanstrup Holm, David Olagnier

**Affiliations:** ^1^Department of Biomedicine, Aarhus Research Center for Innate Immunology, Aarhus University, Aarhus, Denmark; ^2^Fox Chase Cancer Center, Philadelphia, PA, United States

**Keywords:** Nrf2, antiviral immunity, innate immune responses, type I IFN, herpes virus, HSV, STING, genital infection

## Abstract

Herpes simplex virus-2 (HSV-2) is a leading cause of sexually transmitted infections for which no effective vaccines or prophylactic treatment currently exist. Nuclear factor erythroid 2-related factor 2 (Nrf2) is a transcription factor involved in the detoxification of reactive oxygen species (ROS) and has been more recently shown to regulate inflammatory and antiviral responses. Here, we evaluated the importance of Nrf2 in the control of HSV-2 genital infection, and its role in the regulation of HSV-induced innate antiviral immunity. Comparison of antiviral gene expression profile by RNA-sequencing analysis of wild type and *Nrf2*-mutant (*Nrf2*^*AY*/*AY*^) murine macrophages showed an upregulation at the basal level of the type I interferon-associated gene network. The same basal increased antiviral profile was also observed in the spleen of *Nrf2*^−/−^ mice. Interestingly, the lack of Nrf2 in murine cells was sufficient to increase the responsiveness to HSV-derived dsDNA and protect cells from HSV-2 infection *in vitro*. Surprisingly, there was no indication of an alteration in STING expression in murine cells as previously reported in cells of human origin. Additionally, genetic activation of Nrf2 in *Keap1*^−/−^ mouse embryonic fibroblasts increased HSV-2 infectivity and replication. Finally, using an *in vivo* vaginal herpes infection model, we showed that Nrf2 controlled early innate immune responses to HSV-2 without affecting STING expression levels. *Nrf2*^−/−^ mice exhibited reduced viral replication that was associated with higher level of type I interferons in vaginal washes. *Nrf2*^−/−^ mice also displayed reduced weight loss, lower disease scores, and higher survival rates than wild type animals. Collectively, these data identify Nrf2 as a negative regulator of the interferon-driven antiviral response to HSV-2 without impairing STING mRNA and protein expression levels in murine cells.

## Introduction

The Herpesviridae is a large and diverse family of enveloped, double-stranded DNA viruses with relatively complex genomes. Eight of the these herpes viruses are known human pathogens; in particular Herpes Simplex type-1 and−2 (HSV-1 and HSV-2) are leading causes of human viral disease affecting two-thirds of the world's population (1–3). Both of these viruses cause life-long infections due to their ability to establish latency in their hosts with the potential of reactivation. HSV-1 is most often associated with orolabial infection, whereas HSV-2 is generally affiliated with genital ulcers. In rare cases, HSV can cause infection of the central nervous system (CNS) and this can result in herpes simplex encephalitis (HSE) (4–6). Increased acquirement and transmission of human immunodeficiency virus (HIV) is observed in individuals infected with HSV-2 and additionally HSV infections are a major cause of morbidity and occasional mortality in immunocompromised patients (4–6). As no vaccine or prophylaxis exists for HSV-1/2, these agents remain significant public health concerns.

The immune system has evolved different types of early host defense mechanisms to limit viral infection. Toll-Like Receptors (TLRs), primarily present on the cell surface or in endosomal compartments, are involved in the early detection of HSV-derived glycoproteins or nucleic acids in the extracellular milieu (7). These receptors subsequently induce downstream signaling cascades, resulting in potent antiviral responses (7). Critical to sensing HSV in the cytoplasm of infected cells is the cyclic GMP-AMP synthase (cGAS). Upon nucleotide detection, cGAS produces the second messenger, 2′3′-cyclic GMP-AMP (2′3′-cGAMP), which binds to and induces conformational changes in the endoplasmic reticulum adaptor protein stimulator of interferon genes (STING). STING then dimerizes and traffics to the ER-Golgi intermediate compartment (ERGIC), where it recruits the TANK-binding kinase 1 (TBK1), which in turn phosphorylates IFN-regulatory factor 3 (IRF3). IRF3 then translocates to the nucleus as an active dimer that binds specific DNA regions, initiating the transcription of type I IFNs, and other antiviral genes (8, 9). Type I IFNs are secreted cytokines which work in auto- and paracrine manners *via* the IFNα receptor (IFNAR) to upregulate several hundred IFN-stimulated genes (ISGs) that target specific steps in the viral life cycle and inhibit replication. Concurrently, HSV has also evolved multiple strategies to suppress and evade host innate immune responses and facilitate viral infection (10–12). Several HSV gene products are known to counteract the cGAS/STING-mediated DNA sensing pathway. To outline a few, the Virion Host Shutoff (VHS) protein UL41 inhibits cGAS-STING signaling by degrading cGAS via its RNAse activity (13). Additionally, ICP0 and ICP27 can affect the stability and function of STING and inhibit host IRF3 nuclear signaling, thereby preventing type 1 IFN antiviral responses (14, 15).

Nuclear factor-erythroid-2 related factor 2 (Nrf2) is a bZIP transcription factor critical for the production of antioxidant and detoxifying proteins and maintenance of redox homeostasis, particularly in the contexts of stress and infection (16). At steady state, Nrf2 is kept inactive in the cytosol by binding to its repressor Kelch-Like ECH associated protein 1 (Keap1), which licenses it to proteasomal degradation by ubiquitination (17). In response to oxidative stress or electrophilic chemicals, Keap1 is inactivated and Nrf2 is released to freely translocate to the nucleus where it induces the transcription of Nrf2-responsive genes (17). Additionally, Nrf2 was described as an important regulator of the inflammatory response (18) and functions as a transcriptional repressor of inflammatory genes in murine macrophages (19). Moreover, Nrf2 was identified as a target of the immunosuppressive metabolite itaconate (20). In line with these observations, our recent work demonstrated that Nrf2 represses antiviral cytosolic sensing and the generation of type I IFN responses by suppressing the expression of the adaptor protein STING in human cells (21). This inhibition of STING by Nrf2 was sufficient to increase HSV-1 and HSV-2 infectivity in human cells (21).

A wide range of viruses including DENV, Marburg virus, CMV, and HCV have been reported to initiate Nrf2 transcriptional activity either as a result of increased oxidative stress conditions or more directly through the targeting of Nrf2 repressor Keap1 by viral proteins (22–28). Conversely, RSV infection induces a progressive reduction in Nrf2 levels, resulting in a decreased expression of antioxidant gene expression (29, 30). The reasons why some viruses activate Nrf2 while some others repress its antioxidant capacity has yet to be investigated. In the context of HSV-1 infection, murine herpes encephalitis triggered a robust accumulation of ROS in the brain of infected animals, associated with an increased expression of Nrf2-driven antioxidant enzymes, such as HO-1 and Gpx1. Intraperitoneal treatment of the infected animals with the chemical Nrf2 inducer sulforaphane was shown to reduce neurotoxicity and brain inflammation during infection without altering viral replication (31). No studies have yet investigated the role of the Nrf2/Keap1 signaling axis in the control of HSV-2-induced antiviral response and its involvement in the regulation of genital herpes outcome in mice.

In the present work, we demonstrate that Nrf2 suppresses HSV-derived dsDNA-induced antiviral immune responses and increases the susceptibility to HSV-2 genital infection in mice without altering STING expression. Using RNAseq analysis, we show a profound dysregulation of the antiviral gene expression profile between *Nrf2*^*AY*/*AY*^ mutant macrophages, compared to their wild type counterparts. Interestingly, Nrf2 impairs the response to viral-derived dsDNA. Finally, using an experimental murine herpes genital infection model, we demonstrate that Nrf2 deficient animals have an overall better survival rate compared to the WT animals. *Nrf2*^−/−^ mice exhibit reduced viral replication that is associated with higher level of type I IFNs in vaginal washes. Altogether, these findings demonstrate that the Nrf2 transcription factor limits the type I IFN response and increases the susceptibility to HSV-2 vaginal infection in mice.

## Materials and Methods

### Animals

Specific pathogen-free C57BL/6 (WT) mice were bred at Janvier Labs, France and B6.129X1-Nfe212tm1Ywk/J mice (Jackson) were bred at the animal facility, Department of Biomedicine, Aarhus University, Denmark. Animal experimentation was carried out at the animal facility, University of Aarhus. All mice used in this study were age-matched (7–9 week old) female mice on a C57BL/6 background. All described animal experiments have been reviewed and approved by Danish government authorities and comply with Danish laws.

### Primary Cells, Cell Lines, and Culture Conditions

Murine bone marrow-derived macrophages (BMMs) were prepared from bone marrow isolated from femurs and tibiae of C57BL/6 mice. The cells were cultured at a density of 5 × 10^6^ cells/plate, and differentiated over a period of 6 days using RPMI 1640 (Lonza) supplemented with 20–40% L929 cell supernatant (M-CSF producing murine fibroblast cell line), 10% heat-inactivated FCS (Sigma-Aldrich), 10 units/ml penicillin, 10 μg/ml Streptomycin, and 2 mM L-Glutamine (gibco).

Murine peritoneal macrophages were collected by washing peritoneal cavities of mice twice in HBSS. Cells were spun down afterwards and cultured in RPMI containing 10% heat-inactivated FCS (Sigma-Aldrich), 10 units/ml penicillin, 10 μg/ml Streptomycin, and 2 mM L-Glutamine (gibco). WT and *Keap1* KO mouse embryonic fibroblasts (MEFs) were a kind gift of Antonio Cuadrado (Madrid, Spain), and were cultured in DMEM (Lonza) supplemented with 10% heat inactivated fetal calf serum, 200 IU.mL^−1^ penicillin, 100 μg.mL^−1^ streptomycin, and 600 μg.mL^−1^ glutamine.

### dsDNA and cGAMP Stimulation of Cells

HSV-60 naked, a viral dsDNA motif and 2′3′-cGAMP, a STING ligand, were both obtained from Invivogen. Intracellular delivery of dsDNA and cGAMP was achieved using Lipofectamine 2000 (Invitrogen) diluted in serum-free medium with a ratio of Lipo.dsDNA/cGAMP of 1:1. Final concentration for both dsDNA and cGAMP was 4 μg.mL^−1^.

### HSV Production, Quantification, and Infection

HSV-2 333 strain and HSV-2 MS strain were kindly provided by Søren R. Paludan (Aarhus University, Aarhus, Denmark). All HSVs were propagated in Vero cells, purified by ultra-centrifugation, and titrated by standard plaque assay as previously described (32). MEFs, BMMs, or peritoneal macrophages were infected with HSV at the indicated multiplicity of infection (MOI) in a small volume of serum-free medium for 1 h at 37°C. Prior to analysis, cells were incubated with DMEM complete medium containing antibiotics for an additional day of culture.

### Western Blot and Semi-native WB Dimerization Assay

Western blotting was performed as previously reported (21). Antibodies and dilutions used are also mentioned in Olagnier et al. (21). Anti-ICP5 HSV antibody was obtained from (Abcam) (used at 1:3000).

### qPCR Analysis

Gene expression was determined by real-time quantitative PCR, using TaqMan detection systems (Applied Biosciences). For *in vitro* experiments, RNA was extracted using the High Pure RNA Isolation kit (Roche) and RNA quality was assessed by Nanodrop spectrometry (Thermo Fisher). Total RNA from isolated organs (spleen and vagina) was extracted by use of the Qiagen RNase Plus Mini Kit (Qiagen) according to the manufacturer's guidelines and RNA quality assessed by Nanodrop spectrometry (Thermo Fisher). mRNA levels for murine *Ifnb1* (Mm00439552_s1), *Cxcl10* (Mm00445235_m1), *Tmem173* (Mm01158117_m1), *Ifit2* (Mm00492606_m1), *Nfe2l2 (Nrf2)* (Mm00477784_m1), and β*-Actin* (Mm02619580_g1) were analyzed using premade TaqMan assays (Thermo Fisher) and the RNA-to-Ct-1-Step kit according to the manufacturer's recommendations (Applied Biosciences).

### RNAseq Analysis

RNA seq data set was obtained from a previously published manuscript and already available dataset from Otsuki et al. (33). The sequencing reads were aligned to Mm10 genome and the resulting binary alignment (BAM) files were used to calculate the gene counts that represent total number of sequencing reads aligned to a gene. To identify differentially expressed genes between WT vs. *Nrf2*^AY/AY^ samples, DESeq2 algorithm was used (34). The list of differentially expressed genes from DESeq2 output were selected based on 10% adjusted *P*-value level and an FDR of 0.1. Among these, the highly significant genes (FDR <10%) were selected that are involved in pattern recognition signaling, antiviral signaling, and experimentally identified Nrf2 transcriptional targets (21). To depict these genes as a heatmap, count data was transformed using regularized-logarithm transformation (rld) (34), and the resulting values were mean-centered and plotted using pheatmap package available in bioconductor repository [Raivo Kolde (2015). pheatmap: Pretty Heatmaps. R package version1.0.8. http://CRAN.R-project.org/package=pheatmap]. Gene ontology and KEGG pathway enrichment analysis was done using DAVID bioinformatics resources portal (35). Genes involved in antiviral and interferon signaling differentially expressed genes between WT vs. *Nrf2*^*AY*/*AY*^ samples were plotted as functional networks using Ingenuity Pathway Analysis software (QIAGEN Inc., https://www.qiagenbioinformatics.com/products/ingenuity-pathway-analysis).

Human orthologs were depicted after mapping mouse genes using IPA.

### *In vivo* Vaginal HSV-2 Infection

All mice were pretreated by s.c injection of 2 mg of Depo-Provera. Five days later mice were anesthetized with Isoflourane (Sigma-Aldrich) and inoculated intra vaginally with 20 μL HSV-2 (333 strain) (3,33 × 10^5^ PFU) diluted in PBS. To facilitate infection the mice were maintained under anesthesia and placed on their back for 10 min. Vaginal fluids were collected at the indicated time points post infection (p.i.) by pipetting 2 × 40 μL of PBS. The washes were diluted to a finale volume of 250 μL. In selected experiments mice were euthanized by cervical dislocation (c.d.) at different time points and vaginas and spleens were isolated and used for RNA extraction (Qiagen). The virus infected mice were monitored and examined daily and the severity of disease was scored using the following criterias: 0 = healthy, 1 = genital erythema, 2 = moderate genital inflammation, 3 = purulent genital lesion and/or generally bad conditions, 4 = neurological bad conditions and/or severe bad conditions. Mice were sacrificed by c.d. when reaching score 4. For survival studies, Log rank (Mantel-Cox) tests were performed on Kaplan-Meier survival graphs using Prism 7 (GraphPad). Ethical permission was obtained from the Danish Veterinary and Food Administration to perform the vaginal infections. All efforts were made to minimize suffering, and mice were monitored daily during infection.

### Virus Plaque Assay

Virus titer in the collected vaginal fluids were determined on monolayers of Vero cells as previously described (36). Briefly, Vero cells were seeded in DMEM supplemented with 5% FCS at a density of 1.2 × 10^6^ cells/petri dish and left overnight to settle. The next day the cells were infected with diluted samples of the collected vaginal fluids and incubated for 1 h. Subsequently 5 mL of DMEM supplemented with 0.2 % human Immunoglobulin were added and the cells were further incubated for 2–3 days. The cells were stained with 0.03% methylene blue and the plaque were counted.

### Cytokine Measurements

MCP-1 in the vaginal fluids was measured by use of ELISA (R&D development system) according to the manufacturer's instructions. Type 1 IFNs bioactivity was measured by use of an IFN-α/β bioassay based on L929 cells as previously reported (36). UV-light inactivated vaginal fluid samples and IFN-α/β standard was transferred to 96-well plates in successive 2-fold dilutions. Subsequently L929 cells were added to each well and the plates were incubated overnight. The next day Vesicular Stomatis Virus was added to the relevant wells and the plates were incubated for a further 2–3 days. Read out was determined by the dilution mediating 50% protection and defined as 1 Unit of IFN-α/β/mL.

### Statistical Analysis

Values were expressed as the mean ± SEM. Graphs and statistics were computed using Graph Pad Prism 7. An unpaired, two-tailed Student's *t*-test was used to determine significance of the difference between the control and each experimental condition. *P*-values of <0.05 were considered statistically significant, ^***^, *p* < 0.001; ^**^, *p* < 0.01, and ^*^, *p* < 0.05.

## Results

### Nrf2 Dampens Basal Antiviral Gene Levels in Murine Cells

**Figure 1:**
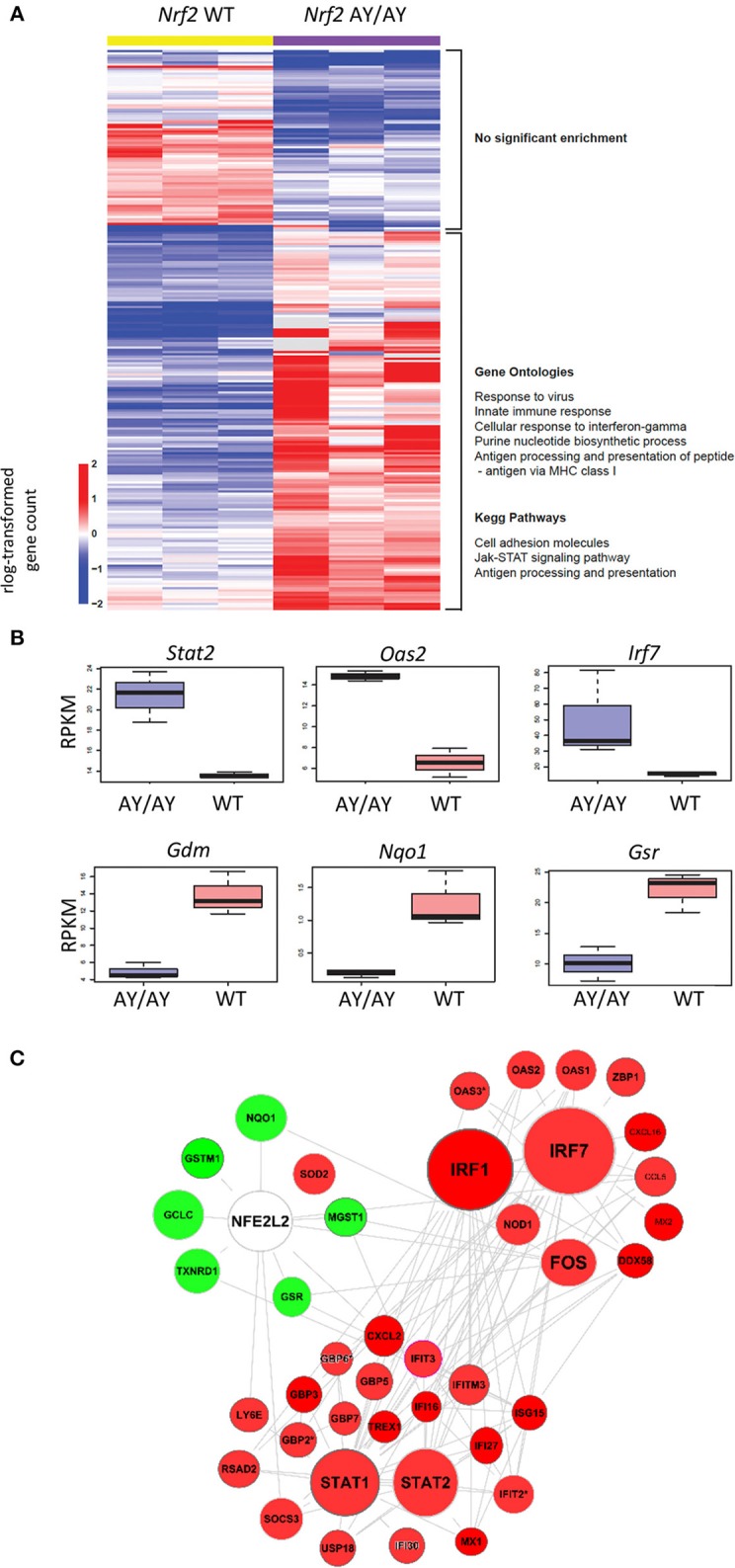
Nrf2 alters the basal level of ISGs in murine macrophages. Comparison of antiviral gene expression profile by RNA-sequencing (RNAseq) analysis of wild-type (WT) and *Nrf2*-mutated (AY/AY) bone marrow-derived macrophages (BMMs). Differentially expressed pathways and genes that satisfied *p*-value <0.001 and fold change cutoff >1 or < -1 were selected. The data are from one experiment performed in triplicate. **(A)** rlog-transformed gene counts and a listing of representative gene ontologies and KEGG pathways associated with these genes is represented in a heatmap. **(B)** Representation of RNA-reads for different interferon-driven and Nrf2-driven genes from RNAseq. Graph displays boxplots with boxes indicating mean, SEM as well as min and max values. **(C)** Cloud analysis of differentially expressed genes from *Nrf2* AY/AY BMMs related to both Nrf2 and IFN-associated pathways is represented. The experiment is a re-analysis of the work of Otsuki et al. (33) (https://www.ncbi.nlm.nih.gov/pubmed/26677805).

To assess the involvement of the transcription factor Nrf2 in the regulation of antiviral gene expression, we analyzed a published RNA-sequencing dataset from Otsuki et al. (33), comparing the transcriptomes of *Nrf2*-mutated AY/AY peritoneal macrophages lacking functional Nrf2 to wild-type (WT) cells. The absolute gene counts were processed and analyzed using DESeq2, which estimates robust expression differences across conditions by considering discreteness, large dynamic range, and the presence of outliers. Since log-transformation of absolute gene counts could result in heteroskedasticity, we used regularized log-transformation of the counts by shrinking the values for genes with higher counts across samples. This step stabilizes the variances across samples. The rlog transformed values for 249 differentially expressed genes are depicted as heatmap. In heatmap, all of the 249 genes that are differentially expressed (Wald-test *p* < 0.001, Benjamini-Hochberg False Discovery Rate <0.06) are shown (Figure 1A). From this analysis, we noticed that pathways related to the innate antiviral immune response, including “Response to Virus, Innate immune Responses, or Cellular Responses to IFN-gamma,” were significantly enriched between WT and *Nrf2*^*AY*/*AY*^ macrophages (Figure 1A). Amongst the differentially expressed genes were several involved in type I IFN signaling and immune responses, including the transcription factors *Irf7* and *Stat2* and the IFN-driven gene *Oas2* (Figure 1B). In sharp contrast, RNA transcripts of known Nrf2-inducible genes, such as *Nqo1, Gsr*, and *Gdm*, were all downregulated in the absence of functional Nrf2 (Figure 1B). To map the interactions across these different genes, a functional clustering node analysis identified a significant intersection between Nrf2 signaling and antiviral-related pathways (Figure 1C). Amongst the most highly induced genes interacting with the Nrf2 antioxidant network were some key transcription factors involved in the early response to viruses including the transcription factors *Irf1* and *Irf7*. Altogether, these data suggest an important link between Nrf2 and the regulation of basal antiviral genes in murine macrophages.

### Nrf2 Impairs Antiviral Gene Expression in Splenic Tissues

**Figure 2:**
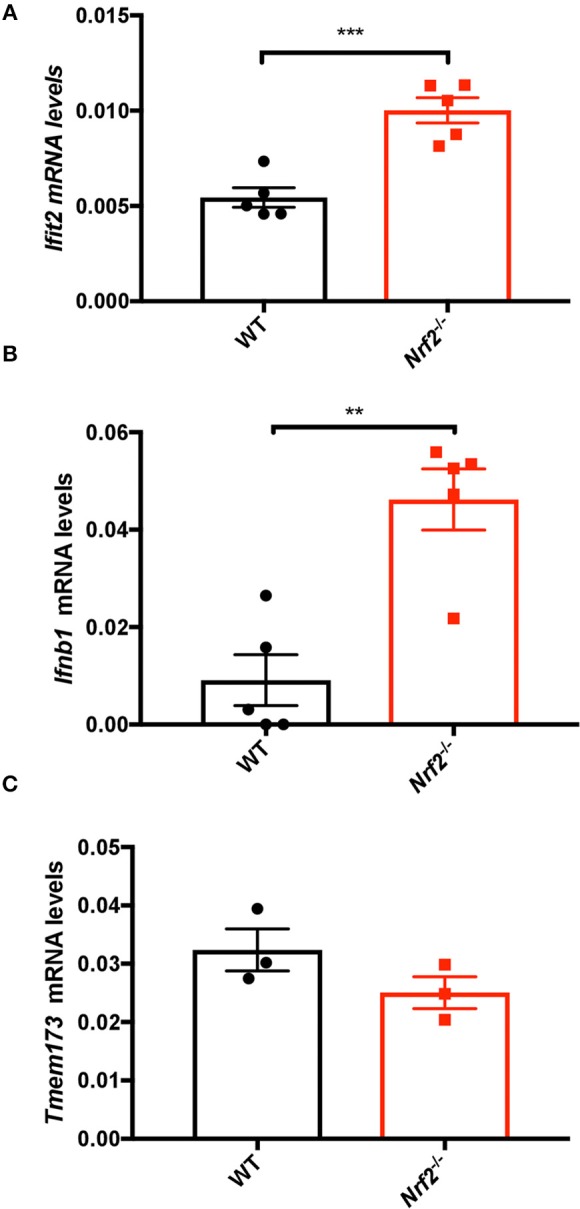
Nrf2 suppresses basal type I IFN and ISG levels in splenic tissues. Spleens from WT and *Nrf2* knockout (KO) mice were harvested and RNA isolated. The basal levels of *Ifit2* (*Isg54*) **(A)**, *Ifn*β*1* **(B)**, and *Tmem173* **(C)** were quantified by qPCR, and normalized to *Actin*. The data are the mean ± SEM where each data point represents one animal.

As Nrf2 repressed antiviral gene expression in murine macrophages, we further asked whether the lack of Nrf2 was also affecting basal antiviral gene levels *in vivo*. To test this hypothesis, spleen tissues from WT and *Nrf2*^−/−^ mice were harvested and RNA isolated for qPCR analysis. The high sensitivity of qPCR gives precise measurements of a sample despite low mRNA level, especially in *ex vivo* studies. Notably, both *Ifit2* (*Isg54*) and *Ifnb1* mRNA levels were significantly heightened in absence of Nrf2 (Figures 2A,B). Surprisingly, STING gene level (*Tmem173*), a critical antiviral adaptor molecule, was unaltered in murine BMMs and in the spleen of *Nrf2*^−/−^ animals compared to WT (Figure 2C and Supplementary Figure 1A), as well as in *Keap1*^−/−^ mouse embryonic fibroblasts (MEFs) (Supplementary Figure 1B). Together, these data argue that Nrf2 may function as a negative regulator of basal antiviral gene expression in murine cells and tissues without altering STING expression.

### Nrf2 Suppresses Innate Antiviral Responses Upon HSV-Derived DNA and cGAMP Stimulation

**Figure 3:**
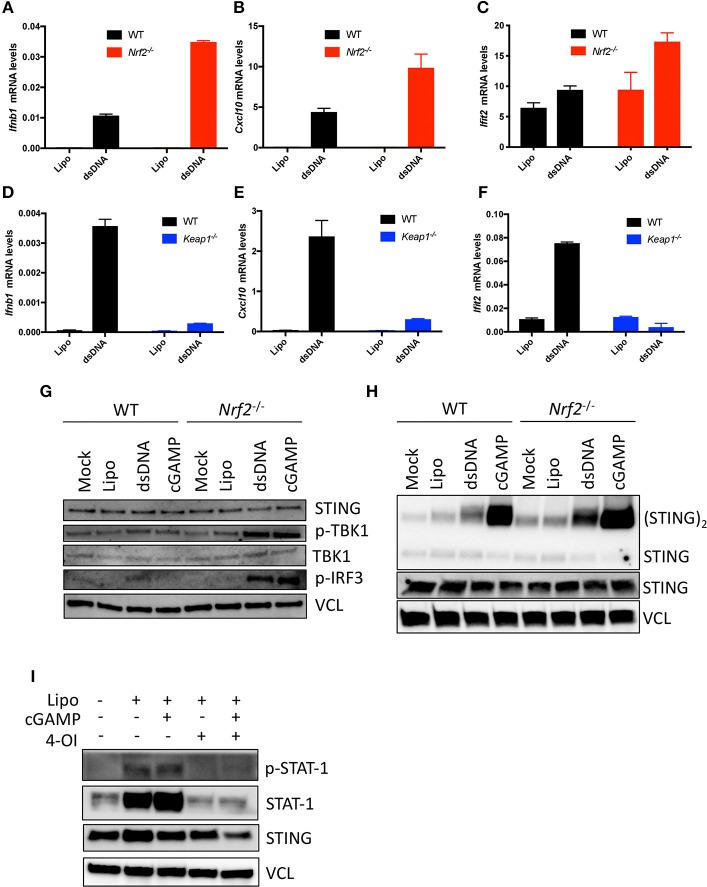
Nrf2 impairs antiviral innate immunity following HSV-DNA or cGAMP stimulation. **(A–C)** WT and *Nrf2* KO BMMs and WT and **(D–F)** *Keap1* KO MEFs were transfected for 6 h with Lipofectamine 2000 (Lipo.) alone or in combination with HSV-derived dsDNA (dsDNA) (4 μg.mL^−1^). RNA was isolated and analyzed for the levels of *Ifn*β*1, Cxcl10*, and *Ifit2* mRNA by qPCR. Data are the means ± SEM where each panel is representative of one experiment performed in duplicate or triplicate. **(G–H)** WT or *Nrf2* KO BMMs were transfected for 3 h with Lipofectamine 2000 alone or in combination with dsDNA or cGAMP (4 μg.mL^−1^) and early antiviral signaling events or STING dimerization were determined by immunoblotting. **(I)** WT MEFs were pre-treated with 4-Octyl-itaconate (4-OI) (125 μM) for ~18 h and then subsequently transfected with Lipofectamine 2000 alone or in combination with cGAMP (4 μg.mL^−1^). Late antiviral signaling events were determined using immunoblotting where vinculin (VCL) is the loading control. The experiments was performed at least twice with similar results.

As Nrf2 repressed antiviral basal gene expression level, we further asked whether Nrf2 was also capable of affecting the responsiveness of cells to activation of the cGAS-STING pathway. WT and Nrf2 deficient BMMs were lipofected for 6 h with either HSV-derived double stranded DNA (HSV-DNA) or the STING ligand, cGAMP, and antiviral gene expression level was assessed by qPCR. The lack of Nrf2 in BMMs resulted in an increased responsiveness to HSV-DNA, as shown by the enhanced expression of *Ifnb1, Cxcl10*, and *Ifit2* mRNA levels in cells deficient for Nrf2 compared to the WT (Figures 3A–C).

Conversely, the induction of *Ifnb1* and the IFN-regulated genes *Cxcl10* or *Ifit2* was significantly reduced in Nrf2 active *Keap1* deficient MEFs following HSV-DNA challenge (Figures 3D–F). To determine the step at which Nrf2 affected the responsiveness of the cGAS-STING pathway in murine cells, *Nrf2*^−/−^ BMMs were stimulated with either HSV-DNA or cGAMP and assessed by immunoblotting for early innate immune signaling events. In response to both DNA and cGAMP, deficiency of Nrf2 affected downstream STING signaling events, as shown by the increased phosphorylation of some of the components in the cGAS-STING pathway TBK1 and IRF3 (Figure 3G). We previously reported Nrf2 to be a suppressor of STING expression in human cells. This did not, however, seem to be the case with murine cells, as no change in STING protein levels could be detected between WT and *Nrf2*^−/−^ BMMs (Figure 3G). We then assessed whether Nrf2 deficiency affected the efficiency of cGAS and STING-dependent events in response to stimulation with dsDNA and cGAMP. Interestingly, STING dimerization was increased in Nrf2 lacking BMMs in response to HSV-DNA and cGAMP compared to WT BMMs (Figure 3H). Finally, we demonstrated that the strength of this Nrf2-dependent antiviral inhibitory response was sufficient to also inhibit downstream STAT1 signaling. Indeed, pre-treatment of WT MEFs with the newly described Nrf2 inducer 4-octyl-itaconate (4-OI) (21) drastically impaired phosphorylation of STAT1 transcription factor in response to cGAMP (Figure 3I).

### Lack of Nrf2 Elevates Type I IFN Response and Decreases Herpes Genital Infection in Mice

**Figure 4:**
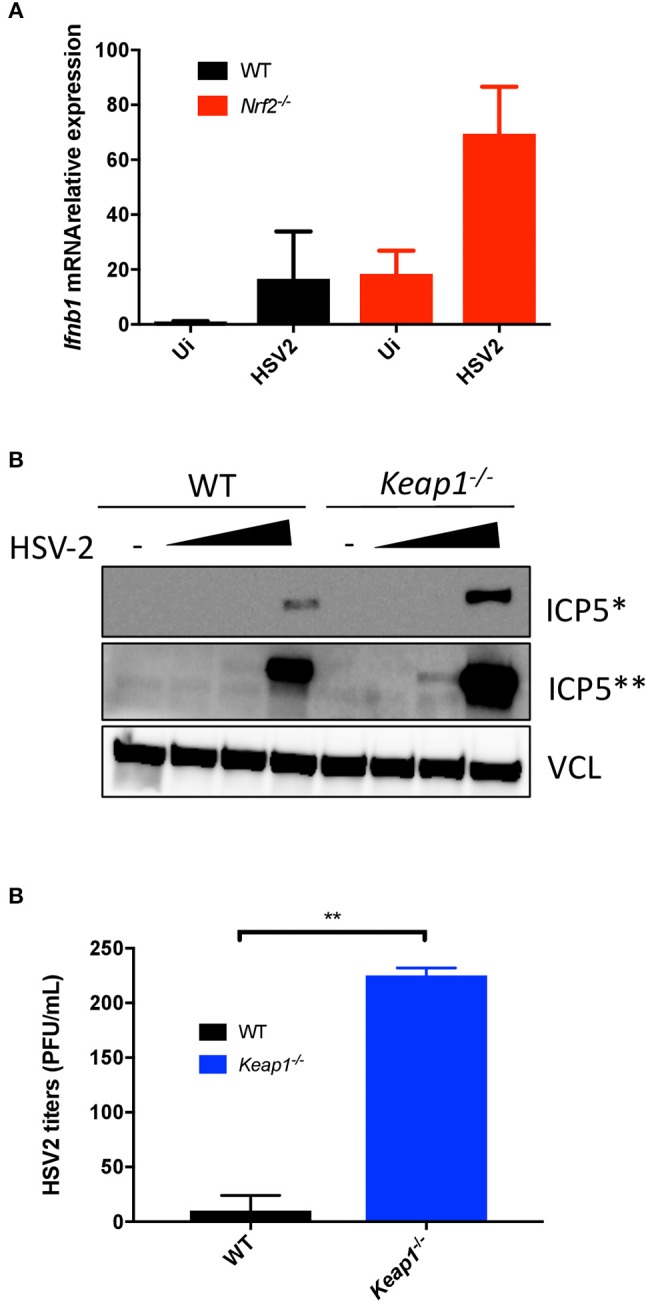
Nrf2 increases the susceptibility to HSV-2 infection *in vitro*. **(A)** WT and *Nrf2* KO peritoneal macrophages were infected with HSV-2, MS strain for 6 h and mRNA level of *Ifn*β*1* was detected by qPCR. **(B)** WT and *Keap1* KO MEFs were infected with HSV-2, MS strain (MOI 0.01, 0.1 and 1, titer 1 × 10^8^ PFU/mL) for ~24 h. ICP5 Viral protein expression was detected by immunoblotting and vinculin (VCL) was used as a loading control. **(C)** The supernatants from samples in **(B)** were used for plaque assay to determine viral replication. The plaque assay was performed on duplicated samples at an MOI of 1.

To further investigate the role of Nrf2 in the control of HSV-2-induced type I IFN and HSV-2 replication, mRNA levels of *Ifn*β*1* were monitored by qPCR following virus infection *in vitro* (HSV-2, strain MS, titer 1 × 10^8^ PFU/mL). Peritoneal macrophages lacking Nrf2 responded more vigorously to HSV-2, as denoted by the ~3-fold increase in *Ifn*β*1* gene expression (Figure 4A). Modulation of HSV-2-induced type I IFN response by Nrf2 was sufficient to repress the expression of HSV-2 ICP5 protein expression in WT MEFs. ICP5 is a major capsid protein of the herpes virus produced in the cytoplasm and translocated to the nucleus upon viral assembly. Conversely, the absence of Keap1 led to an increased accumulation of the viral protein in MEFs (Figure 4B). Finally, we established that genetic activation of Nrf2 through Keap1 deletion promoted HSV-2 replication *in vitro*, as determined by the increased number of replicating virus by plaque assay (Figure 4C), thus implicating Nrf2 in increasing the susceptibility to HSV-2 *in vitro*.

**Figure 5:**
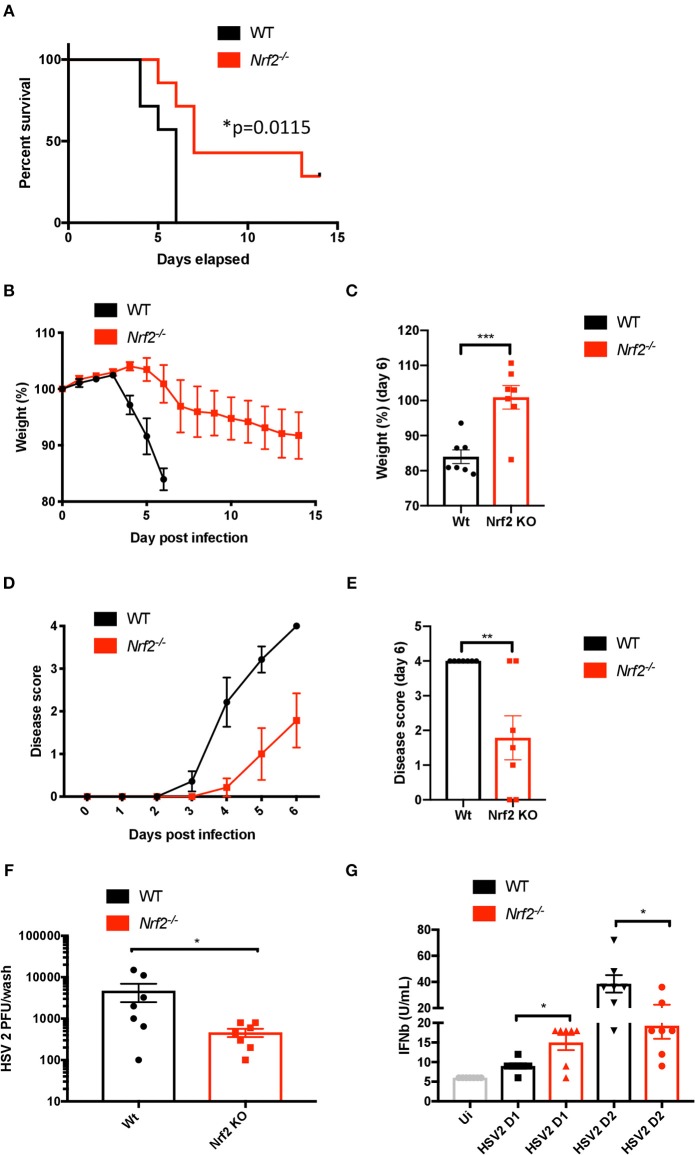
Lack of Nrf2 protects mice from HSV-2 vaginal infection and delays physiopathology. WT and *Nrf2* KO mice were infected intravaginally with HSV-2, strain 333 (3.33 × 10^5^ PFU/mouse). **(A)** Survival, **(B,C)** weight loss and **(D,E)** disease evolution score were measured over time (*n* = 7 mice). **(F)** After 1 day of infection, vaginal washes were collected for use in plaque assay to estimate HSV-2 titers (*n* = 7). **(G)** Type I IFN bioactivity in the vaginal washes was assessed by *in vitro* bioassay (*n* = 7).

To test whether the effect of Nrf2 on the antiviral response is sufficient to affect susceptibility to HSV-2 *in vivo*, we used a herpes genital infection model in mice lacking Nrf2 (infected intravaginally with HSV-2, strain 333, 3.33 × 10^5^ PFU/mouse). Overall, *Nrf2*^−/−^ mice had higher survival rates and reduced weight loss, as well as displayed lower disease scores than their WT counterparts throughout the course of the experiment (Figures 5A–E). Strikingly, the absence of Nrf2 significantly prevented the weight loss observed in WT animals (Figure 5C) and reduced the symptoms of infection after 6 days post-inoculation with the virus (Figure 5E). Additionally, *Nrf2*^−/−^ mice exhibited reduced vaginal viral replication, as determined by plaque assay, that was associated with higher level of type I IFNs in vaginal washes at day 1 post infection (Figures 5F,G). No such increase in chemokine monocyte chemoattractant protein-1 (MCP-1) release, a key regulator in the migration and filtration of monocytes and macrophages, could be observed in the vaginal washes from the same infected animals (Supplementary Figure 2). Furthermore, the increase in type I IFN observed in *Nrf2*^−/−^ animals could not be explained by an enhanced basal level of *Ifnb1*, as seen in the spleen (Figure 2), as no such correlation could be made in the vagina of uninfected WT and *Nrf2*^−/−^ mice (Supplementary Figure 3).

## Discussion

Recent work has shown that Nrf2 is an important regulator of inflammatory responses (18). Moreover, we have previously reported that Nrf2 suppresses the antiviral type I IFN response in human cells (21). However, the link between Nrf2 and the regulation of early innate immune response to virus infections *in vivo* has remained unknown. In the present study, we demonstrated and concluded (i) based on the data of Otsuki et al., that *Nrf2*^*AY*/*AY*^ mutant macrophages displayed a profound dysregulation of the antiviral gene expression profile compared to WT murine cells, (ii) that Nrf2 impaired the response to herpes virus-derived dsDNA without affecting STING expression in murine cells, (iii) that the genetic activation of Nrf2 increased HSV-2 replication *in vitro*, and (iv) that *Nrf2*^−/−^ mice are less susceptible to vaginal infection with HSV-2 due to heightened type I IFN response.

Type I IFNs are known as potent antiviral molecules, and are essential in promoting the expression of ISGs and restricting viral replication (37, 38). Our recent work in human cells demonstrated that Nrf2 restricted type I IFN responses by altering the mRNA stability of the adaptor molecule STING (21).

In the current report, we present data on murine cells, both *in vitro* and *in vivo*, suggesting that Nrf2 also impairs the host antiviral immune response, however, without affecting STING expression. Higher expression of type I IFN and other ISGs were detected in murine cells lacking a functional Nrf2, but no difference in STING level was noticed in murine macrophages (Figure 1). The same observation was made *in vivo*, where the basal levels of ISGs were enhanced in spleens from *Nrf2*^−/−^ animals, but the expression of STING was unaltered (Figure 2). Interestingly, although type I IFN responses are affected by Nrf2 in the mice experiments, the effect of Nrf2 on STING expression seemed to be restricted to human cells. There was no alteration of STING mRNA or protein observed in Nrf2 lacking murine cells. Altogether, this collection of data suggests that Nrf2 uses an alternative way of controlling the antiviral response in murine cells compared with human cells.

Our work demonstrated a dysregulation in the basal levels of type I IFNs and other antiviral associated genes in murine cells mutated or lacking Nrf2. Interestingly, the responsiveness to STING ligands, including cGAMP or HSV-derived dsDNA, was also magnified in absence of Nrf2. Surprisingly, dimerization of STING was enhanced in absence of Nrf2 following cGAMP or dsDNA stimulation, despite no basal change in STING protein level (Figure 3). This observation suggests that Nrf2 could function in controlling STING dimer formation or stabilization. HSV-1 was previously shown to elevate oxidative stress in the cells and trigger S-glutathionylation of TRAF3 and 6 which potentiated PRR signaling (39). As Nrf2 is a master regulator involved in detoxification processes and redox homeostasis in the cell, it is tempting to speculate that either S-gluthationylation or oxidation of STING protein could increase the dimer formation or its stability in absence of Nrf2, and hence potentiates downstream signaling events. Another parameter that could also influence the overall magnified innate immune response to STING ligands observed in absence of Nrf2 is the production of the second messenger cyclic GMP-AMP (cGAMP). Indeed, Cyclic guanosine monophosphate (GMP)–adenosine monophosphate (AMP) synthase (cGAS) is a DNA sensor that triggers innate immune responses through production of cGAMP, which binds and activates the adaptor protein STING (40). Thus, a modulation of cGAS activity by Nrf2 could also lead to an enhanced engagement of STING dimer formation. These different hypotheses will further be tested to uncover the precise mechanistic machinery beyond the effect of Nrf2 on the cGAS-STING pathway in murine cells.

The importance of Nrf2 as a regulator of infection has been previously reported for different viruses (41). For instance, Nrf2-mediated signaling was shown to protect mice against Respiratory syncytial virus (RSV) infection (42). In line with these findings, it was also shown that Nrf2 had a protective role upon Influenza A virus (IAV) and dengue virus (DENV) infection (43, 44). With the DNA virus HSV-2, however, our data suggest that the presence of Nrf2 facilitates HSV-2 replication through the dampening of the antiviral response (Figure 4). Mice lacking Nrf2 exhibited reduced viral replication that was associated with higher level of type I IFNs in vaginal washes. Overall, *Nrf2*^−/−^ mice displayed reduced weight loss, lower disease scores, and higher survival rates than WT animals (Figure 5). Thus, depending on the pathogen and the route of infection, Nrf2 can play either a protective or exacerbating role during host responses to virus infections. Knowledge gained from an *in vivo* model cannot always be translated to humans, and the same is also valid when using murine cells *in vitro* (45, 46). One of the best examples related to STING remains the cleavage of the antiviral adaptor protein by the NS2B3 protease of DENV in human cells, which does not happen in murine or non-human primate cells (47, 48). The use of the herpes genital infection model in mice is based on the investigation of a primary infection with HSV-2. Here, mice are easy to handle, have low costs, and it is a well-described model when it comes to studying herpes genital infection *in vivo* (36). However, this animal model has limitations, including the impossibility of studying recurrent genital infections. In contrast, the guinea pig model provides an excellent tool for studying both primary and recurrent genital herpes infections (49). Additionally, humans share other unique traits with guinea pigs, especially at the immune system level (50). Although findings from our *in vivo* study give a tendency on the possible effect Nrf2 plays in human herpes infection, it is impossible to speculate or predict anything on the outcome Nrf2 might have in a real human vaginal infectious context. For these reasons, expansion of some of our data to a guinea pig model is needed to properly understand the role Nrf2 plays in the course of HSV infection.

In conclusion, our study proposes a model in which Nrf2 transcription factor regulates the early antiviral response to HSV-2. We also unveil the key role of Nrf2 in the physiopathology of HSV-2 vaginal infection, which suggests a possible new therapeutic signaling route to intervene on herpes vaginal infections.

## Data Availability

Sequencing data were previously uploaded dataset to GEO with the following accession number (GSE75176).

## Ethics Statement

The animal study was reviewed and approved by the Danish Veterinary and Food Administration.

## Author Contributions

DO and CH conceived the project, designed the experiments, and supervised the project. CG performed most of the *in vitro* experiments. MI performed all the *in vivo* experiments. SP and SB performed the RNAseq analysis. DO and CG wrote the manuscript. DO assembled the figures. All authors edited and agreed on the submitted version of the manuscript. AT assisted CG in the *in vitro* experiments.

### Conflict of Interest Statement

The authors declare that the research was conducted in the absence of any commercial or financial relationships that could be construed as a potential conflict of interest.
