# Supplementary material for: Nrf2 Negatively Regulates Type I Interferon Responses and Increases Susceptibility to Herpes Genital Infection in Mice
Source: Front Immunol. 2019 Sep 6;10:2101. doi: 10.3389/fimmu.2019.02101 (PMC6742979; doi:10.3389/fimmu.2019.02101)
Supplement: Supplementary file 1 [file Data_Sheet_1.PDF]

A B

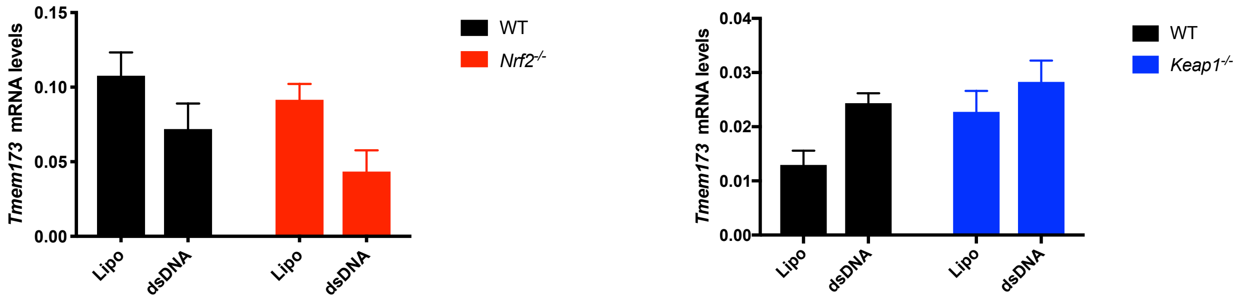

Supplementary Figure 1. *Tmem173* mRNA levels are not altered in Nrf2 deficient or genetically active murine cells. (A) WT and *Nrf2* KO BMMs and **(B)** WT and *Keap1* KO MEFs were transfected for 6h with Lipofectamine 2000 (Lipo.) alone or in combination with HSV-derived dsDNA (dsDNA) ($4\mu.mL^{-1}$). RNA was isolated and analyzed for the levels of *Tmem173* mRNA by qPCR. Data are the means ±SEM where each panel is representative of one experiment performed in duplicate or triplicate.

Suppl. Fig. 1

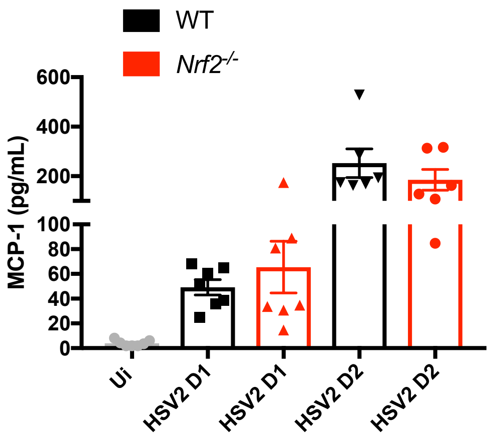

Supplementary Figure 2. MCP1 release following vaginal HSV-2 infection. WT and *Nrf2* KO mice were infected intravaginally with HSV-2, strain 333 ($3.33x10^5$ PFU/mouse). MCP-1 release in the vaginal washes was assessed by ELISA (n=7).

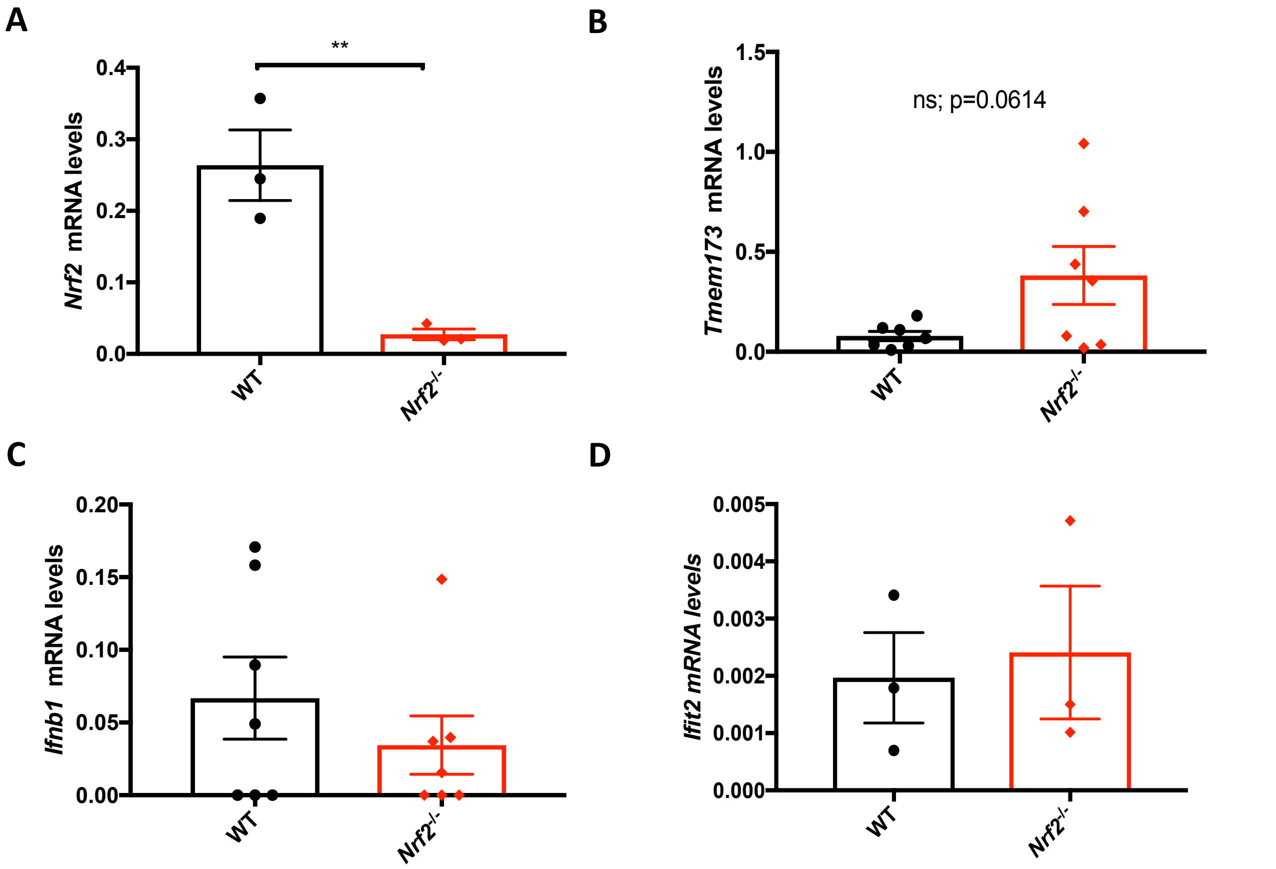

Supplementary Figure 3. Nrf2 does not affect basal type I IFN and ISG levels in vaginal tissues. Vaginal tissue from WT and *Nrf2* knockout (KO) mice were harvested and RNA isolated. The basal levels of *Nrf2* **(A)**, *Tmem173* **(B)**, *Ifnβ1* **(C)** and *Ifit2* **(D)** were quantified by qPCR, and normalized to *Actin*. The data are the mean ±SEM where each data point represents one animal.
